# No Substantial Histopathologic Changes in *Mops condylurus* Bats Naturally Infected with Bombali Virus, Kenya

**DOI:** 10.3201/eid2905.221336

**Published:** 2023-05

**Authors:** Lauri Kareinen, Niina Airas, Sara T. Kotka, Moses M. Masika, Kirsi Aaltonen, Omu Anzala, Joseph Ogola, Paul W. Webala, Olli Vapalahti, Tarja Sironen, Kristian M. Forbes

**Affiliations:** Finnish Food Authority, Helsinki, Finland (L. Kareinen);; University of Helsinki, Helsinki, Finland (L. Kareinen, N. Airas, S.T. Kotka, K. Aaltonen, O. Vapalahti, T. Sironen);; University of Nairobi, Nairobi, Kenya (M.M. Masika, O. Anzala, J. Ogola);; Maasai Mara University, Narok, Kenya (P.W. Webala);; University of Arkansas, Fayetteville, Arkansas, USA (K.M. Forbes)

**Keywords:** Ebola, Bombali virus, *Mops condylurus*, viruses, zoonoses, bat pathology, reservoir host, transmission route, Finland, Kenya

## Abstract

We found similar mild perivascular inflammation in lungs of Bombali virus–positive and –negative *Mops condylurus* bats in Kenya, indicating the virus is well-tolerated. Our findings indicate *M. condylurus* bats may be a reservoir host for Bombali virus. Increased surveillance of these bats will be important to reduce potential virus spread.

Despite extensive research since the first documented human Ebola virus (EBOV) disease outbreak in 1976, animal species involved and mechanisms by which ebolaviruses spillover to humans remains enigmatic. Bats have been implicated as reservoir hosts (*1*), a suspicion supported by several types of evidence, none of which is strong alone. Evidence includes serologic and, rarely, PCR-based diagnosis of infection in wild bats; capacity for some bat species to become asymptomatically infected after laboratory inoculations; and close phylogenetic relationship between ebolaviruses and Marburg virus, which has an established fruit bat reservoir (*2*–*4*). Lack of specimens from naturally occurring ebolavirus-infected bats has precluded assessment of tissue pathology and virus distribution at a cellular level, which would normally provide insights into host roles and transmission mechanisms and permit comparisons and validation of experimental infection models.

We analyzed tissue specimens collected from wild bats in Kenya that were naturally infected with Bombali virus (BOMV), the sixth and most recent ebolavirus identified. First reported in *Mops condylurus* and *Chaerephon pumilus* bats in 2018 (*5*), BOMV has been detected by PCR at low prevalence (0.5%–6.7%) in excreta or tissue samples from *M. condylurus* bats in 4 distinct locations across sub-Saharan Africa (*5*–*8*), representing a consistent ebolavirus–bat host relationship. No further reports of infected *C. pumilus* bats or that BOMV causes human disease have been published (*6*), although prudence is warranted. All other ebolaviruses found in Africa can cause severe human disease (only 1 nonfatal infection with Taï Forest virus has been reported) (*9*). BOMV binds to the same Niemann-Pick C1 endosomal receptor for cell entry as other ebolaviruses (*5*), and recent serologic evidence of a cleared human BOMV infection has been reported (*10*).

## The Study

We captured bats by using mist nets in Busia and Taita-Taveta counties in Kenya during 2019. In brief, we captured, euthanized, and dissected bats and collected tissue samples. We screened the samples for BOMV by using reverse transcription PCR (RT-PCR) as described previously (*8*). We placed bat carcasses that had remaining tissues immediately into 10% buffered formalin, transferred them to 80% ethanol after 24–48 h, and shipped those to the University of Helsinki, Finland, for histopathologic assessment. We analyzed 3 BOMV RT-PCR–positive and 6 BOMV RT-PCR–negative *M. condylurus* bats; the BOMV-negative bats were used as negative controls (*8*).

We trimmed and processed formalin-fixed tissue samples from the 9 bats for histologic evaluation. We routinely stained tissue sections with hematoxylin and eosin. After heat-induced antigen retrieval, we also immunolabeled sections from each tissue sample by using polyclonal rabbit serum against EBOV matrix protein VP40 (*11*) (Appendix). No BOMV isolates were available for this study, which necessitated finding a suitable, cross-reactive surrogate virus. EBOV is the most studied member in the ebolavirus family, and EBOV antibodies are readily available. EBOV and BOMV have ≈75% amino acid identity match within the VP40 protein (Appendix Figure); therefore, we considered EBOV VP40 to be a suitable antigen to detect BOMV VP40.

**Figure 1:**
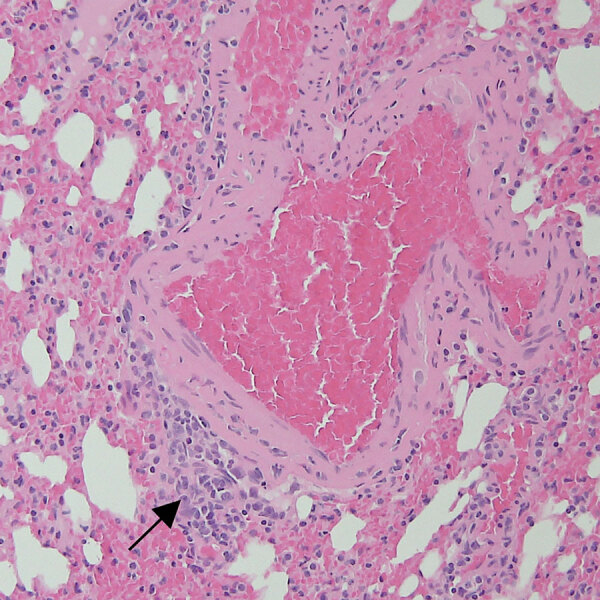
Representative tissue section from the lung of a bat in study of histopathologic changes in *Mops* *condylurus* bats naturally infected with Bombali virus, Kenya. We stained lung tissue sections from a Bombali virus–positive bat with hematoxylin and eosin. Arrow indicates focal minimal mononuclear cell infiltrate. Original magnification ×200.

We observed mild perivascular and interstitial mononuclear infiltrates in all 3 BOMV RT-PCR–positive *M. condylurus* bat lung samples stained with hematoxylin and eosin (Table; Figure 1). We found similar nonspecific mononuclear infiltrates in lung tissue from 4/6 BOMV RT-PCR–negative bats. Overall, we did not observe substantial histopathologic changes in the lungs of any bats that suggested severe acute BOMV disease. We found that both BOMV RT-PCR–positive and RT-PCR–negative bats had mixed acute and chronic inflammatory lesions in other internal organs. Those lesions likely reflect natural disease in wild bats, and we interpreted those findings as incidental. 

**Table:** Lesions found in bat tissues stained with hematoxylin and eosin in study of histopathologic changes in *Mops condylurus* bats naturally infected with BOMV, Kenya*

Organ	Lesion	BOMV-infected†	Uninfected†
Lung	Mild perivascular and interstitial mononuclear infiltrates	3/3	4/6
	Scattered thickening of pulmonary vessel walls	2/3	1/6
	Mild peribronchial mononuclear infiltrates	1/3	2/6
Liver	Mild perivascular mononuclear infiltrates	3/3	1/6
	Moderate chronic neutrophilic infiltrates	0/3	3/6
	Mild hepatocellular vacuolation	0/3	1/6
Heart	Mild mononuclear infiltrates	0/3	2/6
Stomach	Moderate chronic granulomatous gastritis with intralesional nematodes	0/3	2/6

*Comparisons were made between BOMV RT-PCR–positive (BOMV-infected) and RT-PCR–negative (uninfected) *Mops condylurus* bats. Lesions likely represent spontaneous background lesions, except for the neutrophilic infiltrates in liver tissue and granulomatous gastritis nematodiasis. BOMV, Bombali virus. †Number of bats with lesions/total number of bats per group (infected or uninfected).

We did not find an association between immunologic detection of EBOV VP40 antigen, used as a surrogate for BOMV, and overt histopathologic findings in BOMV RT-PCR–positive *M. condylurus* bats. We previously showed that BOMV RT-PCR–positive bats developed antibodies reactive against ebolavirus proteins from Zaire ebolavirus–infected cells (*6*). In this study, both BOMV RT-PCR–positive and RT-PCR–negative bats displayed only mild mononuclear infiltrates in lungs and exhibited mild inflammatory changes in other tissues. Those changes indicate that BOMV infection is likely well-tolerated by *M. condylurus* bats, which is consistent with expectations of a reservoir host (*12*,*13*). However, our findings should be interpreted with caution. For example, we cannot exclude the possibility that the BOMV RT-PCR–positive *M. condylurus* bats we captured had less severe pathology than infected wild conspecifics or that negative control bats might have been previously infected but cleared any residual viral antigens.

**Figure 2:**
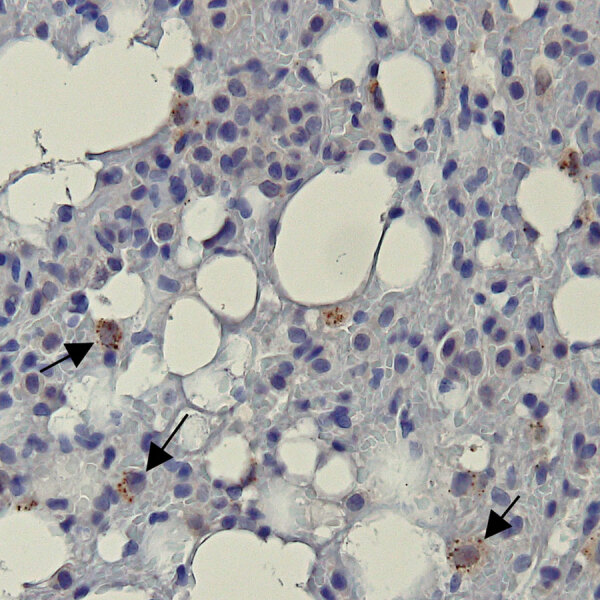
Representative bat lung tissue showing Ebola virus (EBOV) cytoplasmic granules in study of histopathologic changes in *Mops* *condylurus* bats naturally infected with Bombali virus, Kenya. We labeled lung tissue sections by using rabbit polyclonal serum against EBOV matrix protein VP40 and detected antigen by using a chromogenic horse radish peroxidase substrate. The sections were then counterstained with hematoxylin. Arrow indicates granular cytoplasmic immunopositivity for EBOV VP40 antigen. Original magnification ×400. VP, viral protein.

Positive immunolabeling with EBOV VP40 polyclonal antibody was observed in lungs from 2 of 3 BOMV RT-PCR–positive bats (Figure 2) but not in other tissues or in any tissues from BOMV RT-PCR–negative bats. Immunopositive-labeling occurred as granular cytoplasmic aggregates in interstitial macrophages. In contrast to their usual role in protective immunity, macrophages are directly implicated in the pathogenesis of several viruses as either viral repositories or sites of active replication and dissemination (*14*). In humans, pathogenic filoviruses rely heavily on macrophages, which are major replication sites (*15*). Our findings suggest that macrophages might provide similar chronic reservoirs for BOMV infections in bats. However, without data showing active replication, we cannot rule out that our findings in macrophages have a different interpretation.

Positive immunolabeling in bat lungs was consistent with previous RT-PCR results (*8*). Lungs are the most frequent tissue infected by BOMV and have markedly higher viral loads than other tissues (*6*–*8*). Of note, 1 lung sample, 2 spleen samples, and 1 liver sample that were BOMV RT-PCR–positive (*8*) had no detectible EBOV VP40 antigen. Those results are most likely because immunohistochemistry has lower sensitivity than PCR, and a non–BOMV-specific antigen was used.

## Conclusions

We propose that the presence of BOMV in macrophages and absence of acute pathology typical of ebolaviruses (necrosis, thrombosis, hemorrhage, and edema) support bats as a chronic subclinical BOMV reservoir with the potential for infection and sporadic virus excretion. However, the small number of samples, absence of recovery of infectious virus, and lack of longitudinal data make interpretation of our results difficult. As for all wildlife-borne ebolaviruses, no direct evidence currently exists regarding the transmission route of BOMV between susceptible hosts. Our findings suggest the mode of transmission involves activation of replication in either macrophages or other cells and virus dispersal through saliva droplets or feces. The identification of BOMV infection in lungs and occasional virus presence in intestines further supports this hypothesis (*6*–*8*).

In summary, we showed EBOV-VP40 immunolabeling, acting as a cross-reactive surrogate for BOMV antigen, in pulmonary macrophages of wild BOMV RT-PCR–positive *M. condylurus* bats and an absence of substantial concurrent pathologic findings. Our results support *M. condylurus* bats as reservoir hosts for ebolaviruses and highlight the importance of bat surveillance to understand and mitigate potential emerging disease risks.

Appendix Additional information for no substantial histopathologic changes in *Mops condylurus* bats naturally infected with Bombali virus, Kenya.
