# Supplementary material for: No Substantial Histopathologic Changes in Mops condylurus Bats Naturally Infected with Bombali Virus, Kenya
Source: Emerg Infect Dis. 2023 May;29(5):1029–32. doi: 10.3201/eid2905.221336 (PMC10124661; doi:10.3201/eid2905.221336)
Supplement: Appendix — Additional information for no substantial histopathologic changes in Mops condylurus bats naturally infected with Bombali virus, Kenya. [file 22-1336-Techapp-s1.pdf]

Article DOI: https://doi.org/10.3201/eid2905.221336

EID cannot ensure accessibility for supplementary materials supplied by authors. Readers who have difficulty accessing supplementary content should contact the authors for assistance.

No Substantial Histopathologic Changes in *Mops condylurus* Bats Naturally Infected with Bombali Virus, Kenya

Appendix

IHC protocol

Antigen retrieval was performed in citrate buffer (pH 6). Tissue sections were prepared on slides and incubated for 20 min at a maximum temperature >99°C during antigen retrieval. After antigen retrieval, the sections were washed with 1× Tris-buffered saline (TBS) for 5 min and then incubated in 3% hydrogen peroxide for 10 min. Next, the sections were washed 2 × 5 min each in 1× TBS and then incubated in 10% bovine serum albumin for 20 min. Rabbit polyclonal antiserum Ag204 against Ebola virus matrix protein VP40 (*11*, main text) was diluted 1:500 in Animal Free Blocker and Diluent (Vector Laboratories, https://www.vectorlabs.com) and added to the tissue sections. Sections were incubated for 60 min with the primary antibody and then washed 2 × 5 min each in 1× TBS. For each sample, a negative control was similarly prepared; the primary antibody was replaced with a rabbit isotype control diluted at 1:500. Secondary antibody (BrightVision anti-rabbit HRP 1 step detection system) (Immunologic, http://www.immunologic.nl) was added to the sections and incubated for 30 min. The sections were washed 2 × 5 min each in 1× TBS. Bright DAB chromogen (Immunologic) was added to the sections and incubated for 6 min; the slides were then rinsed in water. Sections were counterstained with hematoxylin (Papanicolaou's solution 1a Harris hematoxylin solution, Sigma-Aldrich, https://www.sigmaaldrich.com) for 10 s and rinsed with tap water for 3 min.

```
CLUSTAL O(1.2.4) multiple sequence alignment


tr|A0A0D5W8L9|A0A0D5W8L9_9MONO      MRRVILPTAPPEYMEAIYPARSNSTIARGGNSNTGFLTPESVNGDTPSNPLRPIADDTID      60
tr|A0A4D5SGV0|A0A4D5SGV0_9MONO      MRRTVIPTAPPDYTEALYPQRANSIGSTSTSTNQQYYPTDVPGGDIPSNSLRPVADDNID      60
                                    ***.::*****:* **:** *:**  : . .:*  :   :  .** *** ***:***.**

tr|A0A0D5W8L9|A0A0D5W8L9_9MONO      HASHTPGSVSSAFILEAMVNVISGPKVLMKQIPIWLPLGVADQKTYSFDSTTAAIMLASY      120
tr|A0A4D5SGV0|A0A4D5SGV0_9MONO      HTNHTPDNVSSAFILEAMVNVISGPKVLMKQIPIWLPLGVADQKKYSFDSTTAAIMLASY      120
                                    *:.***..************************************.***************

tr|A0A0D5W8L9|A0A0D5W8L9_9MONO      TITHFGKATNPLVRVNRLGPGIPDHPLRLLRIGNQAFLQEFVLPPVQLPQYFTFDLTALK      180
tr|A0A4D5SGV0|A0A4D5SGV0_9MONO      TITHFGKTSNPLVRVNRLGPGIPDHPLRLLRLGNQAFLQEFVLPPVQLPQYFTFDLTALK      180
                                    *******::**********************:****************************

tr|A0A0D5W8L9|A0A0D5W8L9_9MONO      LITQPLPAATWTDDTPTGSNGALRPGXSXHPKLRPILLPNKSGKKGNSADLTSPEKIQAI      240
tr|A0A4D5SGV0|A0A4D5SGV0_9MONO      LITQPLPAATWTDEVLLTSPNMLRPGLSFHPKLRPILLPGKPGKKGANLGLTAPDKIHAI      240
                                    *************:.   * . **** * **********.* **** . .**:*:**:**

tr|A0A0D5W8L9|A0A0D5W8L9_9MONO      MTSLQDFKIVPIDPTKNIMGIEVPETLVHKLTGKKVTSKNGQPIIPVLLPKYIGLDPVAP      300
tr|A0A4D5SGV0|A0A4D5SGV0_9MONO      MDHLQDLKVVPIDSSKNIVGIEVPDALVQKLTGKKPHAKNGQSIIPVLLPKYIGPDPVSA      300
                                    *  ***:*:**** :***:*****::**:******  :**** *********** ***:

tr|A0A0D5W8L9|A0A0D5W8L9_9MONO      GDLTMVITQDCDTCHSPASLPAVVEK  326
tr|A0A4D5SGV0|A0A4D5SGV0_9MONO      SDLTMVITQDCDTCNSPASFPIASEK  326
                                    .*************:****:* . **
```

Appendix Figure. Alignment of Ebola virus (UniProt no. A0A0D5W8L9) and Bombali ebolavirus (UniProt no. A0A4D5SGV0) VP40 matrix proteins. We used the UniProt protein database (https://www.uniprot.org) align-tool and found ≈75% amino acid identity between the 2 VP40 proteins. Asterisks represent fully conserved residues between sequences, colons represent conservation between amino acids with strongly similar properties, and periods represent conservation between amino acids with weakly similar properties.
